# Investigating intermittent immersion during osmotic dehydration of mango (*Mangifera indica* L. Moench). Part A: Determination of optimal conditions for mango (*Mangifera indica* L. Moench) dehydration impregnation by immersion (D2I) and intermittent immersion (D3I)

**DOI:** 10.1016/j.heliyon.2024.e35808

**Published:** 2024-08-13

**Authors:** C. Tsopwo Zena, Y. Jiokap Nono

**Affiliations:** a Department of Process Engineering, National Advanced School of Agro-industrial Sciences, ENSAI, Ngaoundere University, P.O. Box 455, Ngaoundere, Cameroon; b Department of Chemical Engineering and Environment, University Institute of Technology, IUT, Ngaoundere University, P.O. Box 455, Ngaoundere, Cameroon

**Keywords:** Mango, Dehydration impregnation by immersion, Intermittent process, Optimization, Solute gain, Water loss

## Abstract

This work aimed to determine the optimum conditions for dehydration impregnation by immersion (D2I) and by intermittent immersion (D3I) of mango (*Mangifera indica*) slices measuring 4 × 1 × 1 cm^3^. To this end, the Doehlert response surface plan was used, with the following factors for D2I: the volume of D2I solution/fruit mass ratio (6/1–13/1 mL/g), the process time (120–360 min) and the Brix degree of the solution (45–65 °Brix) and with the following factors for D3I: immersion time (20–60 min), process time (60–300 min) and de-immersion time (7–25 min). The temperature was fixed according to literature at 35 °C. The optimum responses obtained for the D2I process were (47.63 ± 1.79) g/100 g (w-b) for water loss, and (6.67 ± 1.04) g/100 g (w-b) for solute gain, for optimum operating conditions of 6/1 mL/g; 245 min and 61.6°Brix respectively for the immersion ratio, process time and solute concentration of the hypertonic solution. The optimum responses obtained for D3I process were (47.98 ± 2.12) g/100 g (w-b) for water loss, and (4.31 ± 0.052) g/100 g (w-b) for solute gain (SG), for operating conditions of 21; 270; and 9 min, respectively for immersion time, process time and de-immersion time. The Student's t-test on the predicted and experimental optima of WL and SG revealed valuable insights for comparing these two processes. The present study will undoubtedly introduce a new dynamic to the osmotic dehydration systems for fruits and vegetables.

## Highlights

• A low solution volume/mass ratio and a high concentration of hypertonic solution favor water loss and prevent solute gain.
• V/m ratio and solution concentration low or high produce high solute gain.
• Immersion and de-immersion times are significant for solute gain at the 5 % significant level.
• The less the immersion and de-immersion times, the less the solute gain and the more the water loss.
• The solute gain obtained after D3I is equal to 2/3 of the solute gain obtained in D2I.

## 1 Introduction

Mango (*Mangifera indica*) is a member of the Anacardiaceae family, which includes 73 genera and around 850 species [1]. It essentially consists of four parts namely: the stalk, the exocarp (skin), the mesocarp, and the endocarp (core) [1,2]. The exocarp of the fruit is thick and glandular. On the other hand, its mesocarp, rich in fiber, carbohydrates, vitamin C, beta-carotene, polyphenols, and trace elements, varies in thickness depending on the variety and is resinous and highly variable in terms of flavor, shape, size, color, and fiber content [3,4]. Mango is eaten ripe or green as a dessert or salad and is an important part of the diet in many African countries [5]. In addition to its good organoleptic and nutritional properties, mango has a very high water activity (0.97–0.99), making it highly perishable. Several preservation techniques have been used to prevent post-harvest losses during the harvest period, including dehydration (drying), which is one of the most important and widely used.

Drying consists of eliminating the moisture impregnating a material by evaporation till the desired moisture level, which is not dangerous for its conservation over a longer period [6]. However, during this operation, the coupled mass and heat transfers induce physical and biochemical changes, thereby altering organoleptic properties [[7], [8], [9]]. Moreover, the drying process consumes a significant amount of energy, particularly because drying fruits with high water content typically requires approximately 5000 kJ/kg of evaporated water [10]. To avoid these various losses, researchers recommend the application of pre-treatments before drying [[11], [12], [13]]. Of all the pre-treatments used before drying, osmotic dehydration is the only one that allows fruits to be pre-dehydrated. Osmotic dehydration is a simple pre-treatment to implement and consists of immersing a material in a hypertonic solute solution (sugar, salt, etc.). During immersion, the liquid (water + soluble substances) present in the cellular materials of the biological material (generally fruit and vegetables) will diffuse into the hypertonic solution, and simultaneously, the solute present in the hypertonic solution will impregnate the surface of the biological material and gradually migrate inwards [[14], [15], [16]]. Due to the solute impregnation that occurs during osmotic dehydration, this operation is still called dehydration impregnation by immersion (D2I) [17]. The criteria used to evaluate this pre-treatment are water loss (WL) and solute gain (SG). WL and SG depend on both the intrinsic properties of the biological material (porous structure, size, shape, surface area of the product) and the treatment operating conditions (time, treatment temperature, pressure, solution agitation, nature of the solute, concentration of the hypertonic solution and sample mass/hypertonic solution volume ratio) [18,19].

Unfortunately, the mass transfer rate during the osmotic dehydration of plant foods is generally slow; the cellular membrane exerts high resistance to transfer and slows down the osmotic dehydration rate [18,20]. Thus, there has always been a need to develop supplementary techniques to enhance the mass transfer without adversely affecting the quality of plant foods. Therefore, the partial damage of cell membranes using different pre-treatment methods can be advantageous for the acceleration of mass transfer processes. These pre-treat methods before osmotic dehydration include an edible coating [21,22], freeze and thawing [23,24], ultrasound [[25], [26], [27]], high hydrostatic pressure [28,29], pulsed electric fields [[30], [31], [32], [33]], pulsed vacuum [34,35], centrifugal force [35], microwave heating [36,37], ohmic heating [38,39], gamma irradiation [40], skin pretreatment [41,42]. These techniques added to OD not only increase the complexity of the process, which is intended to be simple to implement but also increase the cost of the process. They also increase the rate of water loss as well as the rate of solute gain [20]. A high solute gain is not always desirable for organoleptic or nutritional reasons (as in the case of diabetics), but it also acts as a major brake on the dehydration process during subsequent drying.

Considering the operational factors that affect D2I, certain aspects have not been thoroughly studied to fully grasp their impact on mass transfers during OD. In addition, no optimal V/m ratio has been proposed for the D2I of mango, which has an intrinsic configuration and resistance to mass transfer that differ from one fruit and vegetable to another. In several works on OD, the V/m ratio is generally set to a value, between 4/1 and 60/1 mL/g including that of 100/1 mL/g [18,20]. As dehydration progresses, the hypertonic solution becomes increasingly diluted, and therefore, for a specific fruit, the V/m ratio must be carefully chosen so that the driving force for water elimination is still sufficient at the end of the process [18]. Depending on the process variables, the amount of the diffusing solute is generally around 5–20 % of the initial mass of the fruit. This quantity alters the composition and final taste of the fruit [43] and contributes to blocking the surface layers, providing additional resistance to mass transfer during the OD operation [44]. Therefore, finding an ideal V/m ratio for mango OD is crucial to achieving the lowest SG and highest WL.

Optimization studies on osmotic dehydration show that the scientific challenge of minimizing solute gain during OD remains unchanged. With this in mind, this study proposes a technique that will provide an additional solution for minimizing solute gain during OD. Of all the work from the literature presented on D2I, we note that immersion is always continuous throughout the process, with variations in terms of agitation [45,46]. At present, several works using intermittence have been developed in several transformation processes to improve mass transfers in biological materials. In the case of drying, intermittence has been developed to reduce energy costs but also to improve transfers and thus limit crusting phenomena for products rich in carbohydrates [[47], [48], [49], [50]].

For the moment, the dynamics of mass transfer during discontinuous immersions have not been the subject of scientific studies, at the level of the D2I process, whereas intermittence could contribute to minimizing solute gain, which is the main objective sought to reduce crusting problems during subsequent drying operations. Dehydration Impregnation by Intermittent Immersion (D3I) will therefore consist of interrupting temporarily immersion of a biological material in a hypertonic solute solution.

This study will aim to understand the effect of factors on water loss and solute gain during D2I and D3I and to find the optimum processing conditions for D2I and D3I to maximize water loss and minimize solute gain.

## 2 Material and methods

### 2.1 Preparation of raw materials

**Fig. 1:**
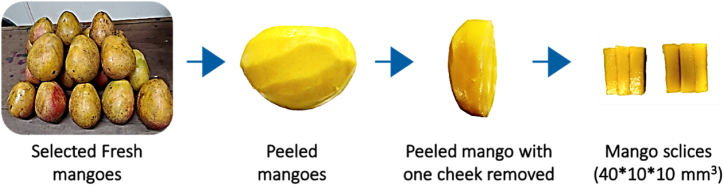
Steps to obtain the mango slices.

The mango (*Mangifera indica*) used in this study is the variety locally known as "coffee mango". This variety is the most widely produced, marketed, and consumed in the Vina department of the Adamawa region of Cameroon [51]. The mangoes, at the same level of maturity and showing no signs of deterioration, were bought at the local market (Dang) in Ngaoundere III and taken to the laboratory for storage before the experiments began. Before each experiment, 40x10 × 10 mm^3^ mango parallelepiped were obtained following the steps described in Fig. 1.

### 2.2 Preparation of the hypertonic solution

**Fig. 2:**
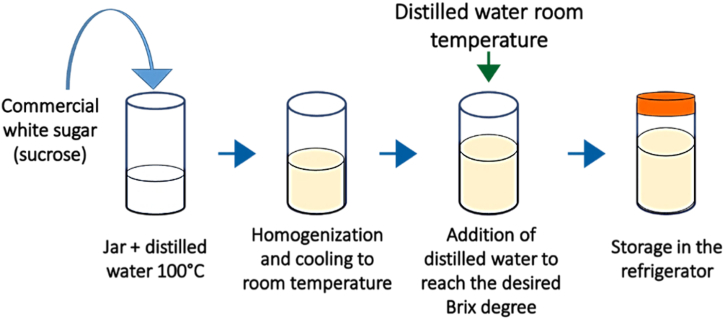
Step in the production of hypertonic sucrose solution.

The hypertonic solutions were prepared using an appropriate quantity of sucrose (food grade, purchased at the local market in Ngaoundere III) and distilled water in proportion to the desired hypertonic solution concentration. Sucrose was used as the sole solute because of its lower cost, accessibility, dehydrating power, and organoleptic properties [52,53]. Hypertonic solutions were prepared according to the steps described in Fig. 2. The hypertonic solution sucrose concentrations were assessed at room temperature using a HANNA HI 96801 digital refractometer with a reading range of 0–85 %.

### 2.3 Dehydration impregnation by immersion (D2I) of mango slices

#### 2.3.1 Procedure

**Fig. 3:**
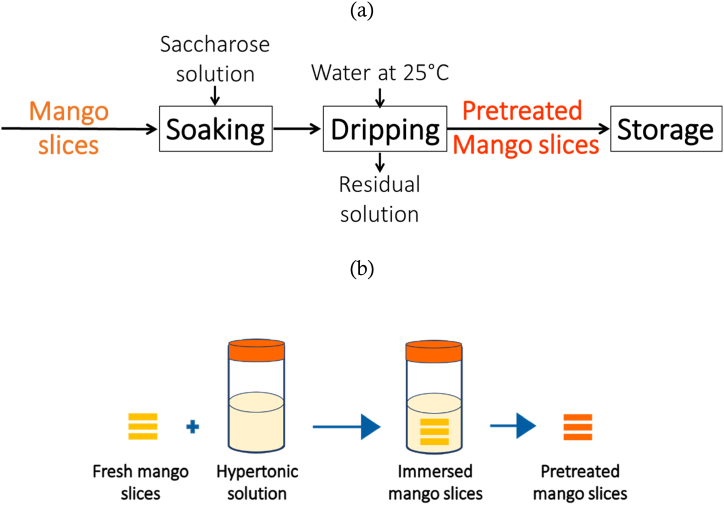
Process (a) and operating steps (b) for obtaining mango slices D2I-pretreated.

D2I of mango slices was carried out in glass jars with lids, placed inside an incubator (Incucell 111, Medcenter Einrichtungen GmbH D-82166 Gräfelfing/Germany, made by MMM-Group). D2I was carried out at 35 °C using the process and steps described in Fig. 3.

After each experiment, the mango slices were removed from the hypertonic solution, drained, rinsed with very little water, drained a second time, and weighed. The water content of the mango slices pretreated with D2I was then determined to calculate water loss (WL) and solute gain (SG). Material transfer between the hypertonic solution and the mango slices during D2I was evaluated using parameters such as water loss (WL) and solute gain (SG) according to equations (1), (2) respectively.

(1) $$\text{WL}=\frac{M_{0}\cdot X_{w0}-M_{t}\cdot X_{\text{wt}}}{M_{0}}$$

(2) $$\text{SG}=\text{WL}-\frac{M_{0}-M_{t}}{M_{0}}$$

With M_0_: sample mass, (g), at time t = 0; M_t_: sample mass, (g), at time t; X_wo_: weight fraction of water, (g/g w-b) at time t = 0; X_wt_: weight fraction of water, (g/g w-b), at time t.

#### 2.3.2 Experimental design for D2I

Response surface methodology was used to evaluate the effects of factors on WL and SG during D2I of mango slices. The V/m ratio (X_1_), D2I process time (X_2_), and Brix degree (X_3_) were selected as independent variables using literature studies and preliminary experiments. Table 1 shows the uncoded values of the selected factors from the literature.

**Table 1:** Coded and uncoded values of the selected factors for the D2I process.

Factors	Level (−1)	Level (−0.5)	Level (0)	Level (−0.5)	Level (+1)
V/m ratio (X_1_) in (mL/g)	6/1	7.75/1	9.5/1	11.25/1	13/1
Process time (Tps_Proces, X_2_) in (min)	120	180	240	300	360
Hypertonic solution concentration (X_3_) in (°Brix)	45	50	55	60	65

Of the three optimization designs proposed by the response surface methodology, the Doehlert design was used to design the experimental data because it allows to obtaining a second-degree model with fewer trials. Additionally, this design allows for varying levels between factors, enabling us to prioritize one factor over another based on the specific information we seek to obtain from the study. With these three factors, Doehlert's design gave us 15 experiments. Each experiment in the design was repeated three times. The values of both coded and uncoded factors are presented in Table 2. The transition from coded to real (uncoded) values was performed using equations (3), (4):

(3) $$X_{i}=x_{i}\cdot \text{Pas}+X_{0}$$

(4) $$\text{Pas}=\frac{\left(X_{+1}-X_{-1}\right)}{2}$$

With Pas: the increment step; X_i_: the actual value of factor i; x_i_: the coded value of factor i; X_+1_: the actual value of factor i at level +1; X_-1_: Actual value of factor i at level −1 and X_0_: Value at the center point (level 0).

**Table 2:** Experimental matrices for the D2I process using the Doehlert design.

n°	Coded values			Uncoded values		
	x_1_	x_2_	x_3_	X_1_	X_2_	X_3_
1	0	0	0	9.5/1	240	55
2	1	0	0	13/1	240	55
3	0.5	0.866	0	11.25/1	344	55
4	−0.5	0.866	0	7.75/1	344	55
5	−1	0	0	6.1/1	240	55
6	−0.5	−0.866	0	7.75/1	136	55
7	0.5	−0.866	0	11.25/1	136	55
8	0	0	0	9.5/1	240	55
9	−0.5	0.289	0.816	7.75/1	275	63.2
10	0.5	−0.289	−0.816	11.25/1	205	46.8
11	−0.5	−0.289	−0.816	7.75/1	205	46.8
12	0.5	0.289	0.816	11.25/1	275	63.2
13	0	−0.577	0.816	9.5/1	171	63.2
14	0	0.577	−0.816	9.5/1	309	46.8
15	0	0	0	9.5/1	240	55

### 2.4 Dehydration impregnation by intermittent immersion (D3I) of mango slices

#### 2.4.1 Procedure

The D3I operation was carried out in batch mode, in jars with lids, placed in an

incubator (INCUCELL 111, Medcenter Einrichtungen GmbH D-82166 Gräfelfing/Germany, made by MMM-Group). D3I was carried out at 35 °C under the optimum conditions of V/m ratio and concentration of the hypertonic of the D2I process in the present work.

**Fig. 4:**
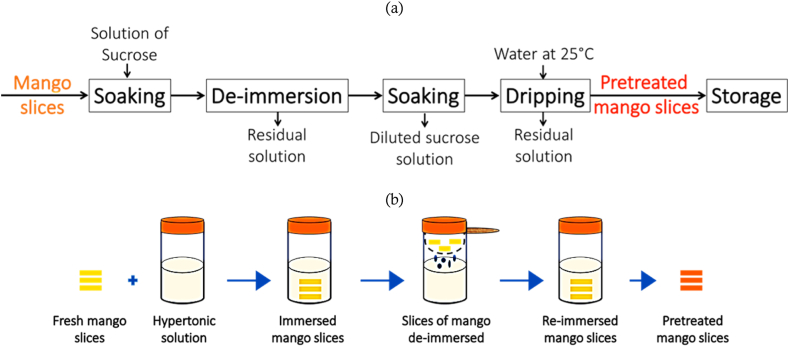
Process (a) and operating steps (b) for obtaining mango slices pretreated with the D3I process.

The process and operating steps described in Fig. 4 show how this operation is carried out. During the D3I operation, immersion in the hypertonic solution was interrupted for a specific period (to be determined) at specific immersion time intervals (to be determined) until the end of the dehydration process.

After each experiment, the mango slices were removed from the hypertonic medium, drained, rinsed with very little water, drained a second time, and weighed. The water content of the mango slices, pretreated with D3I, was then determined to calculate water loss and solute gain. Material transfer between the hypertonic solution and the mango slices during D3I was evaluated using parameters such as water loss (WL) and solute gain (SG) according to equations (1), (2) respectively.

#### 2.4.2 Experimental design for D3I

Response surface methodology was used to assess the main effects of factors on water loss (WL) and solute gain (SG) during D3I of mango slices. Immersion time (X_1_ or Tps_Im), D3I process time (X_2_ or Tps_Proces), and de-immersion time (intermittency time) (X_3_ or Tps_De-Im), were selected as independent variables using preliminary experiments. Table 3 shows the uncoded values of various factors chosen from the literature.

**Table 3:** Coded and uncoded values of the selected factors for the D3I process.

Factors	Level (−1)	Level (−0.5)	Level (0)	Level (−0.5)	Level (+1)
Immersion time (Tps_Im, X_1_) in min	20	30	40	50	60
Process time (Tps_Proces, X_2_) in min	60	120	180	240	300
De-Immersion time (Tps_De-Im, X_3_) in min	7	11.5	16	20.5	25

Similar to the D2I process, the Doehlert design was employed here, resulting in 16 experimental trials. Table 4 presents the coded and uncoded values of the factors. The transition from coded values to real values was carried out using equations (3), (4). Each experiment in the design was repeated three times.

**Table 4:** Experimental matrices for the D2I process using the Doehlert design.

	Coded values			Uncoded values		
n°	x_1_	x_2_	x_3_	X_1_	X_2_	X_3_
1	0	0	0	40	180	16
2	1	0	0	60	180	16
3	0.5	0.866	0	50	283.92	16
4	−0.5	0.866	0	30	283.92	16
5	−1	0	0	20	180	16
6	−0.5	−0.866	0	30	76.08	16
7	0.5	−0.866	0	50	76.08	16
8	0	0	0	40	180	16
9	−0.5	0.289	0.816	30	214.68	23.344
10	0.5	−0.289	−0.816	50	145.32	8.656
11	−0.5	−0.289	−0.816	30	145.32	8.656
12	0.5	0.289	0.816	50	214.68	23.344
13	0	−0.577	0.816	40	110.76	23.344
14	0	0.577	−0.816	40	249.24	8.656
15	0	0	0	40	180	16
16	0	0	0	40	180	16

### 2.5 Statistical analysis

Statistical analysis of the experimental results was performed using Minitab Statistical Software 21 (Copyright © 2022 Minitab, LLC. All rights reserved) and Design Expert 13 (Copyright © 2021. All rights reserved). The postulated model chosen for the experiment is the quadratic model (equation (5)) which presents three parts: the simple or linear effect part (β_i_x_i_), the quadratic effect part (β_ii_x_i_^2^), and the interaction effect part (β_ij_x_i_x_j_).

(5) $$Y=\beta_{0}+\sum_{i=1}^{3}\beta_{i}x_{i}+\sum_{i=1}^{3}\beta_{\text{ii}}x_{i}^{2}+\sum_{j=2}^{3}\sum_{i=1}^{j-1}\beta_{\text{ij}}x_{i}x_{j}+\in$$

With x: is the coded independent variable; β_0_, β_i_, β_ii_, β_ij_: are constant regression coefficients, and **ε**: total error which is the difference between the observed values and the estimated values of the response.

The contribution of the linear, quadratic, and interaction terms of each model was calculated according to equations (6), (7), (8).

■ Contribution of linear terms:

(6) $$\text{Contribution}\;\left(\%\right)=\frac{\left|\beta_{i}\right|}{\sum_{i=1}^{k}\left|\beta_{i}\right|+\sum_{i=1}^{k}\left|\beta_{\text{ii}}\right|+\sum \sum_{i≺j}^{k}\left|\beta_{\text{ij}}\right|}$$

■ Contribution of quadratic terms:

(7) $$\text{Contribution}\;\left(\%\right)=\frac{\left|\beta_{\text{ii}}\right|}{\sum_{i=1}^{k}\left|\beta_{i}\right|+\sum_{i=1}^{k}\left|\beta_{\text{ii}}\right|+\sum \sum_{i≺j}^{k}\left|\beta_{\text{ij}}\right|}$$

■ Contribution of interaction terms:

(8) $$\text{Contribution}\;\left(\%\right)=\frac{\left|\beta_{\text{ij}}\right|}{\sum_{i=1}^{k}\left|\beta_{i}\right|+\sum_{i=1}^{k}\left|\beta_{\text{ii}}\right|+\sum \sum_{i≺j}^{k}\left|\beta_{\text{ij}}\right|}$$

The models, obtained through multiple linear regression analysis, were evaluated by measuring the statistical parameters presented in Equations (9), (10), (11), (12), (13), with validation criteria provided in Table 5. Additionally, lack-of-fit statistics were analyzed to assess any significant discrepancies between the model and the observed data, at the 5 % significance level. Once the models had been validated, the significant terms in the model were determined by analysis of variance (ANOVA) for each response. Significance was assessed at the 5 % threshold.

(9) $$R^{2}=1-\frac{\sum_{i=1}^{N}{\left(Y_{\left(i,\exp\right)}-Y_{\left(i,\text{calc}\right)}\right)}^{2}}{\sum_{i=1}^{N}{\left(Y_{\left(i,\exp\right)}-{\bar{Y}}_{\left(i,\exp\right)}\right)}^{2}}$$

(10) $$R_{\text{adj}}^{2}=1-\frac{\left(1-R^{2}\right)\cdot N-1}{N-p}$$

(11) $$\text{AADM}=\frac{\sum_{i=1}^{N}\left(\frac{\left|Y_{\left(i,\exp\right)}-Y_{\left(i,\text{calc}\right)}\right|}{Y_{\left(i,\exp\right)}}\right)}{N}$$

(12) $$B_{f}=10^{\frac{1}{N}\sum_{i}^{N}\log\left(\frac{Y_{\left(i,\text{calc}\right)}}{Y_{\left(i,\exp\right)}}\right)}$$

(13) $$A_{f}=10^{\frac{1}{N}\sum_{i}^{N}\left|\log\left(\frac{Y_{\left(i,\text{calc}\right)}}{Y_{\left(i,\exp\right)}}\right)\right|}$$

with R^2^: coefficient of determination; R^2^_a__d__j_: adjusted coefficient of determination; AADM: Absolute analysis of deviation from the mean; B_f_: Bias factors; A_f_: Accuracy factors. Y_i,exp_: experimental response; Y_i,calc_: predicted response; p: number of constants and N: number of observations.

**Table 5:** Recommended values for statistical criteria ranges for model validation.

Cricteria	Standard values	Acceptable values	References
R^2^ and R^2^_adj_	1	> 80 %	[54]
AADM	0	[0–0.3]	[55]
B_f_ and A_f_	1	[0.75–1.25]	[56]

### 2.6 Optimization using the desirability function

Regarding Response Surface Methodology, the optimization of multiple responses was performed by using the desirability functions. The advantages of using desirability functions include the following: (i) responses that have different scaling can be compared, (ii) the transformation of different responses to one measurement is simple and quick, and (iii) both qualitative and quantitative responses can be used [57,58]. It is based on the idea that the quality of a product or process that has multiple quality characteristics, with one of them outside of some desired limits, is completely unacceptable. The method finds operating conditions x that provide the most desirable response values [59]. Depending on whether a particular response Y_i_ is to be maximized or minimized, equation (14) or 15 are used, respectively [56,57,59].

(14) $$d_{i}\left(Y_{i}\right)=\left\{ \begin{array}{c} 0\\ {\left(\frac{Y_{i}\left(x\right)-L_{i}}{T_{i}-L_{i}}\right)}^{S}\\ 1 \end{array}\right.\;\begin{array}{c} Y_{i}\left(x\right)<L_{i}\\ L_{i}\leq Y_{i}\left(x\right)\leq T_{i}\\ Y_{i}\left(x\right)>T_{i} \end{array}$$

(15) $$d_{i}\left(Y_{i}\right)=\left\{ \begin{array}{c} 1\\ {\left(\frac{Y_{i}\left(x\right)-U_{i}}{T_{i}-U_{i}}\right)}^{S}\\ 0 \end{array}\right.\;\begin{array}{c} Y_{i}\left(x\right)<T_{i}\\ T_{i}\leq Y_{i}\left(x\right)\leq U_{i}\\ Y_{i}\left(x\right)>U_{i} \end{array}$$

where L_i_, Ui, and T_i_ are the lower, upper, and target values, respectively, that are desired for response Y_i_, s is a shaping parameter of the desirability. For s = 1, the desirability function increases linearly towards T_i_; for s < 1, the function is convex, and for s > 1, the function is concave [56].

After desirability values are computed for each response variable, they are combined into a single desirability index, D, by calculating their geometric mean. Further refinements to the weighting can be applied by assigning a range of numbers to the importance of optimizing each response variable. The final desirability index then is computed as presented in equation (16) [59]:

(16) D=(d1vi×d2vi×d3vi×⋯⋯×dnvi)1∑vi=(∏i=1ndivi)1∑vi

where v_i_ is a number indicating the relative importance of the ith response, which might typically be an integer in the range of 1–5, with 5 indicating the greatest importance and 1 indicating the least.

In the present study, the maximization and minimization of the fitted polynomials were carried out using the desirability function found in Minitab Statistical Software 21. For this study, the optimization criteria were maximization of the WL and minimization of the SG. The best optimal factors should present a desirability close to 1.

After determining the optimal operating conditions, experiments were conducted using the model's predicted optimal conditions. A Student's t-test was then performed to evaluate the validation of the predicted optimal responses.

### 2.7 Experimental validation of the predicted optimal responses

We employed a *t*-test statistical method to determine the significant difference between optimal responses observed in experimental results and those predicted. Initially, we assessed the equality of variances (homoscedasticity) of the two groups using an F-test before conducting a two-tailed test. The 'F-test' function in Excel was utilized, providing a probability (p-value) indicating whether the variances in the two groups of data significantly differ. A p-value greater than the significance level suggests homoscedasticity; otherwise, we assume unequal variances [58]. Based on the result of the F-test, we conducted a *t*-test at a 5 % significance level. This involved calculating either a pooled or an unpooled t-statistic to compare the means $\bar{x_{1}}$ and $\bar{x_{2}}$ of two samples with sizes $n_{1}$ and $n_{2}$ , and sample standard deviations $s_{1}$ and $s_{2}$ , respectively. The pooled t-statistic (equation (17)) is used when the variances between the two samples are assumed to be equal. The pooled standard deviation can then be calculated as presented in equation (18). Conversely, the unpooled t-statistic (Welch's *t*-test) is applied when the variances are considered separate in the case of two independent samples (equation (19)). In this case, the degree of freedom for calculating the t-critical value is given in equation (20) (Satterthwaite's formula) [58]. According to Montgomery (2017) [58], using the appropriate formula based on the variance assumption ensures that the statistical test is robust and the conclusions drawn are valid [58].

(17) $$\text{pooled}\;t.\text{statistic}=\frac{\left|\bar{x_{1}}-\bar{x_{2}}\right|}{s_{pooled}\cdot \sqrt{\frac{1}{n_{1}}+\frac{1}{n_{2}}}}$$

where $s_{pooled}$ is the pooled standard deviation calculated as:

(18) $$s_{pooled}=\sqrt{\frac{\left(n_{1}-1\right)\cdot {s_{1}}^{2}+\left(n_{2}-1\right)\cdot {s_{2}}^{2}}{n_{1}+n_{2}-2}}$$

(19) $$\text{unpooled}\;t.\text{statistic}=\frac{\left|\bar{x_{1}}-\bar{x_{2}}\right|}{\sqrt{\frac{{s_{1}}^{2}}{n_{1}}+\frac{{s_{2}}^{2}}{n_{2}}}}$$

(20) $$df=\frac{\left(\frac{{s_{1}}^{2}}{n_{1}}+\frac{{s_{2}}^{2}}{n_{2}}\right)}{\frac{{\left(\frac{{s_{1}}^{2}}{n_{1}}\right)}^{2}}{n_{1}-1}+\frac{{\left(\frac{{s_{2}}^{2}}{n_{2}}\right)}^{2}}{n_{2}-1}}$$

The *t*-test was conducted under the null hypothesis (H0) that there is no statistically significant difference between the predicted and experimental responses, with the alternative hypothesis (H1) stating a significant difference. Microsoft Excel version 16.0.17628.20144 was used to compute the t-critical value and the p-value of the t-statistic for the two-tailed test. If the calculated t-value is lower than the t-critical value, we do not reject the null hypothesis; if higher, we reject it. Failure to reject the null hypothesis occurred when the *t*-test p-value exceeded the significance level. Individual t-tests were conducted for each response.

To estimate the overall prediction error of the model, we exploited the concepts of residual variance and mean square error provided by the ANOVA table. The 95 % confidence interval for the predictions ($\hat{y}$) was calculated according to equation (21).

(21) $$IC=\hat{y}\pm t_{\propto /2,n-p}\cdot SEP$$

where IC is the confidence interval, $t_{\propto /2,n-p}$ is the critical value of the Student's t-distribution, (n-p) is the degree of freedom of the residual for a 95 % confidence level, with n being the number of trials and p being the number of estimated parameters, including the intercept. SEP is the standard error of prediction, defined in equation (22). SSR is the sum of squares of residuals (referred to as Error in the ANOVA table).

(22) $$SEP=\sqrt{\frac{SSR}{\left(n-p\right)}}$$

Regarding the optimal experimental responses, results were presented as (value±standarddeviation).

## 3 Results and discussion

### 3.1 Doehlert design results for mango slice treatment using the D2I process

#### 3.1.1 Experimental results, model expressions, and validation for the D2I process

The experimental values of water loss (WL) and solute gain (SG), for the D2I process, are presented in Table 6. From this table, it can be seen that the WL varies from (30.58 ± 0.51) % to (54.22 ± 0.50) % and the SG varies from (4.64 ± 1.02) % to (9.46 ± 0.78) % depending on the operating conditions provided by the experimental matrix. Based on these results, we observe that certain operating conditions achieve a WL of 50 % and an SG of 4 %, which meets our expectations considering our target of 50 % WL and 5 % SG.

**Table 6:** Experimental values of water loss (WL) and solute gain (SG), for the D2I process based on the Doehlert design.

Essay	Factors			WL		SG	
	X_1_	X_2_	X_3_	Exp	SD	Exp	SD
1	9.5/1	240	55	42.633	0.519	8.320	0.519
2	13/1	240	55	44.707	0.684	7.642	0.684
3	11.25/1	344	55	47.869	1.135	9.299	1.427
4	7.75/1	344	55	47.290	0.485	8.381	0.743
5	6/1	240	55	41.489	0.429	6.859	0.583
6	7.75/1	136	55	30.583	0.510	6.754	0.581
7	11.25/1	136	55	33.273	1.329	7.614	1.179
8	9.5/1	240	55	41.455	1.402	7.706	1.626
9	7.75/1	275	63.2	54.224	0.496	7.661	0.313
10	11.25/1	205	46.8	37.521	0.947	6.991	1.296
11	7.75/1	205	46.8	34.557	0.799	8.584	0.974
12	11.25/1	275	63.2	51.221	0.692	9.463	0.785
13	9.5/1	171	63.2	40.794	1.321	4.641	1.017
14	9.5/1	309	46.8	39.022	1.682	7.802	0.952
15	9.5/1	240	55	42.074	0.835	8.656	1.662

With Exp: experimental; SD: standard deviation and X_1_, X_2_, and X_3_ real values of the factors respectively the V/m ratio, the process time, and the Brix degree.

The analysis of variances presented in Table 7, enabled us to determine the coefficients of the second-degree model hypothesized for the experimental designs. The WL and SG models, with coded values for factors, are presented in equations (23), (24):

(23) $$Y_{\text{WL}}=42.054+1.208x_{1}+10.312x_{2}+8.791x_{3}+1.044x_{1}^{2}-4.554x_{2}^{2}+2.773x_{3}^{2}-1.408x_{1}x_{2}-3.951x_{1}x_{3}+6.728x_{2}x_{3}$$

(24) $$Y_{\text{SG}}=8.227+0.444x_{1}+1.484x_{2}-0.404x_{3}-0.977x_{1}^{2}+0.052x_{2}^{2}-1.237x_{3}^{2}+0.038x_{1}x_{2}+2.535x_{1}x_{3}+4.419x_{2}x_{3}$$

**Table 7:** ANOVA of polynomial models predicting water loss (WL) and solute gain (SG) of mango slices in the D2I process.

Source	df	WL				SG			
		SCar	Contri (%)	Coef	P value	SCar	Contri (%)	Coef	P value
Model	9	594.533		42.054	0.000	17.5597		8.227	0.052
**Linear**	3	530.603	49.820	/	0.000	7.829	20.125	/	0.038
Ratio	1	5.839	2.963	1.208	**0.024**	0.789	3.832	0.444	0.227
Tps_Proces	1	318.961	25.294	10.312	**0.000**	6.606	12.805	1.484	**0.010**
°Brix	1	205.593	21.563	8.791	**0.000**	0.435	3.488	−0.404	0.354
**Quadratic**	3	27.222	20.533	/	0.005	1.767	19.546	/	0.342
Ratio^2^	1	1.308	2.561	1.044	0.190	1.145	8.429	−0.977	0.158
Tps_Proces^2^	1	13.995	11.170	−4.554	**0.004**	0.002	0.444	0.052	0.950
°Brix^2^	1	4.536	6.802	2.773	**0.037**	0.902	10.672	−1.237	0.201
**Interaction**	3	23.565	29.648	/	0.007	8.727	60.330	/	0.031
Ratio*Tps_Proces	1	1.115	3.454	−1.408	0.220	0.001	0.327	0.038	0.967
Ratio*°Brix	1	6.228	9.691	−3.951	**0.021**	2.563	21.872	2.535	**0.056**
Tps_Proces*°Brix	1	13.550	16.503	6.728	**0.005**	5.845	38.131	4.419	**0.013**
**Error**	5	2.842	/	/	/	2.082	/	/	
Lack of fit	3	2.147	/	/	0.343	1.618	/	/	0.315
Pure error	2	0.695	/	/	/	0.464	/	/	
**Total**	**14**	**597.375**	**100.00**	**/**	**/**	**19.642**	**100.00**	**/**	

With df: degree of freedom; Coef: Coefficient; Contri: contribution and Lack of fit: Inadequacy of the fit; SCar: sum of squares of deviations.

Table 7 presents p-values for the lack-of-fit tests of 0.343 and 0.315 for WL and SG, respectively. Since these p-values are greater than the significance level of 0.05, we can accept the null hypothesis of no lack-of-fit in both models. Table 8 displays the validation statistics for WL and SG. For the SG model ((Y_SG_)), the criteria for R^2^, AADM, B_f_, and A_f_, fall within the acceptable validation range, although the adjusted R^2^ does not. In contrast, all statistical criteria for the WL model are validated.

**Table 8:** Statistical values for the validation of WL and SG models in the D2I process.

Responses	R^2^	R^2^ adj	AADM	B_f_	A_f_
**Y**_**SG**_	89.40	70.32	0.042	1.002	1.042
**Y**_**WL**_	99.52	98.67	0.009	1.001	1.009

Attempts to find a better-fitting model for SG yielded less satisfactory results: the cubic model produced aliased p-values for lack of fit, the linear model had p-values below the significance level of 0.05, and the Two-Factor Interaction (2FI) model showed lower R^2^ and adjusted R^2^ values compared to the quadratic model. Similarly, other models for WL (linear, 2FI, and cubic) provided inferior results.

Consequently, these results indicate that the quadratic model best fits the experimental data for both WL and SG. This conclusion is based on the absence of significant lack-of-fit, the highest R^2^ and adjusted R^2^ values, and favorable bias and accuracy factors. Considering the degrees of freedom for lack of fit, pure error, and total correlation, the WL and SG models reliably explain the variability in their respective experimental data, thereby validating the models.

With these findings, it is crucial to assess the effects of the various factors on the responses.

#### 3.1.2 Study of the significance and contribution of factors for the D2I process

The significance of the factors for each response was determined through an analysis of variance. The impacts of these factors on the responses, along with their contributions and coefficients, are detailed in Table 7. In the case of WL, the contribution of factors with a simple effect is 49.82 % (almost 50 %), factors with a quadratic effect contribute 20.53 %, and factors with an interaction effect contribute 29.65 %. This indicates that individual factors significantly influence WL during the D2I process, and interactions between factors further enhance the WL process. For SG, the contribution of factors with a simple effect is 20.13 %, factors with a quadratic effect contribute 19.55 %, and factors with an interaction effect contribute 60.33 % (more than 50 %). This suggests that interactions between factors have a greater influence on SG during the D2I process than simple or quadratic effects. Specifically, the time-Brix and ratio-Brix interactions have a significant impact on SG.

Table 7 shows that time and its quadratic effect, Brix and its quadratic effect, the interaction between Brix and the ratio, and time and the ratio are significant at the 5 % threshold for WL. Additionally, time, Brix, the ratio, the time-Brix interaction, and the quadratic effect of Brix positively affect WL, whereas other factors have a negative effect. Notably, the V/m ratio is not only statistically significant at the 5 % threshold but increasing its value during D2I increases WL. This underscores the importance of carefully selecting the ratio as a factor in hypertonic dehydration processes, considering that many scientific studies arbitrarily fix it.

The interaction between ratio and Brix, as well as time and its interaction with hypertonic solution concentration, are significant at the 5 % significance level and positively affect SG, as shown in Table 7. These findings align with those reported in the literature, demonstrating that the longer a biological material is immersed in a high concentration of solute, the more it absorbs solute until equilibrium is established [18,20,60].

#### 3.1.3 Singular effects of factors on responses during the D2I process

##### 3.1.3.1 Singular effects of factors on water loss (WL) during the D2I process

**Fig. 5:**
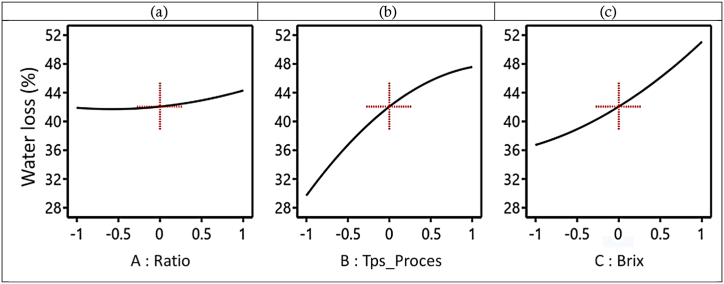
Evolution of WL as a function of V/m ratio (a), process time (b), and Brix degree (c) during the D2I process.

Fig. 5 shows the evolution of WL as a function of the V/m ratio, D2I process time, and Brix degree. Fig. 5 (a) shows the evolution of the WL as a function of the ratio. We note that the WL increases slightly, from 42 to 45 g of water/100 g (w-b), with an increase in the ratio from −1 to 1 (6/1 to 13/1 mL/g). We can see at first glance that varying the ratio does not improve the WL for a process time of 240 min and a Brix of 55°Brix. It can be observed that working with high ratios in D2I isn't beneficial. Not only does it complicate the process and necessitate bulky containers compared to using a low ratio, but it also requires more solute to achieve a Brix of 55°Brix compared to a low ratio, which achieves practically the same WL. This observation is illustrated in Fig. 5(a).

In Fig. 5 (b), we observe an increase in the WL with the increase in the process time from 27 to 47 g of water/100 g (w-b) for a time ranging from −1 to 1 (120–360 min). In Fig. 5 (c) we observe that the WL increases, from 37 to 51 g of water/100 g (w-b), with the increase in the Brix of the hypertonic solution from −1 to 1 (45–65°Brix), and all this for a ratio of 9.5/1 g/mL. This allows us to note, as do several studies presented in the literature, that the longer a biological material remains immersed in a hypertonic solution of increasing concentration, the more it will lose water and the more it will gain solute until thermodynamic equilibrium is reached.

##### 3.1.3.2 Singular effects of factors on solute gain (SG) during the D2I process

**Fig. 6:**
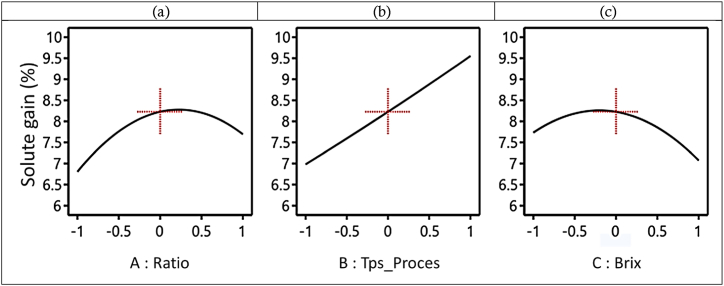
Evolution of SG as a function of V/m ratio (a), process time (b), and Brix degree (c) during the D2I process.

Fig. 6 shows the evolution of SG as a function of the V/m ratio, D2I process time, and Brix degree. Fig. 6(a) shows the evolution of WL as a function of the ratio. From this figure we can observe that the SG increases from 6.75 to 8.25 g solute/100 g (w-b) before decreasing from 8.25 to 7.5 g solute/100 g (w-b) for a ratio ranging from −1 to 1 (6/1 to 13/1 mL/g), this for 240 min, in a hypertonic solution of 55°Brix. It comes out that the SG is low at low ratios and high at high ratios, in the range of tested values. In Fig. 6 (b), for immersion in a hypertonic solution with a ratio of 9.5:1 mL/g and a concentration of 55 °Brix, we observe an increase in SG from 7 to 9.5 g of solute/100 g (w-b) with an increase in process time from −1 to 1 (120–360 min). This observation has been made by several authors in literature reviews where it is shown and demonstrated that the longer a biological material remains immersed in a hypertonic solute solution, the more it becomes impregnated with the solute until saturation occurs.

In Fig. 6 (c), for immersion in a hypertonic solution with a ratio of 9.5/1 mL/g for 240 min, we observe an overall decrease in SG, from 8 to 7g solute/100 g (w-b), with an increase in Brix ranging from −1 to 1 (45–65 °Brix). This observation contrasts with findings from authors in the field as demonstrated in literature reviews, where it is shown that higher Brix levels of hypertonic solutions lead to increased impregnation of solute into biological materials. Therefore, in principle, we expect SG to increase with time under these conditions. From this study, we can note that the influence of the V/m ratio on the WL and the influence of the V/m ratio and the Brix on the SG have evolutions that do not present the conventional evolutions predicted by the bibliographical reviews in the field. For deeper insights into how the V/m ratio and Brix interact with WL and SG, an interaction curve is crucial.

#### 3.1.4 Effects of the ratio-Brix interaction on responses during the D2I process

##### 3.1.4.1 Effects of the ratio-Brix interaction on water loss (WL) during the D2I process

**Fig. 7:**
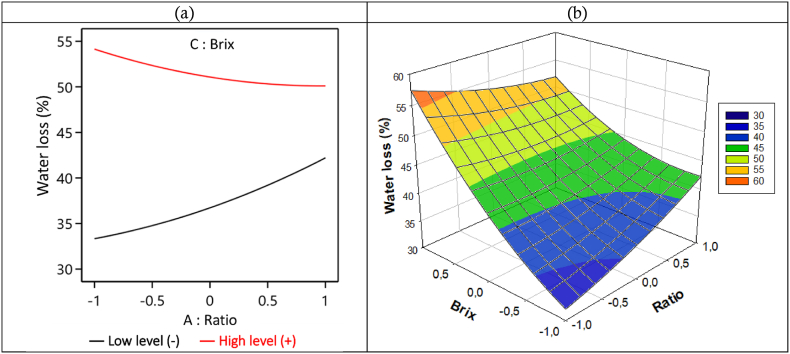
Interaction curve V/m ratio – Solution Brix degree on WL in 2D (a) and 3D (b), during the D2I process.

Fig. 7 shows the effect of the interaction between the V/m - Brix ratio on the WL during 240 min of immersion. We can see that when the Brix level is low (−1: 45 °Brix), the WL increases from 33 to 42 g of water/100 g (w-b) with the increase in the ratio from −1 to 1 (6/1 to 13/1 mL/g). But when the Brix is at its highest level (1: 65 °Brix) we see instead a slight decrease in the WL from 54 to 50 g water/100 g (w-b) with the increase in the ratio. From this analysis, we can note that the WL is at its highest when the ratio is at its lowest (6/1 mL/g) and the Brix at its highest (1: 65 °Brix). We then realize that when Brix is high, WL decreases with increasing ratio, whereas at low Brix, WL increases with increasing ratio. However, in the literature, authors typically focus on the influence of the concentration of the hypertonic solution on mass transfers, often overlooking the impact of the ratio (volume of the solution versus the mass of the sample) [24]. Our present work demonstrates that this parameter significantly determines the final result.

Firstly, it should be noted that the driving force of the hypertonic solution, which enables the removal of water from the biological material, is high when the Brix level is high and low when the Brix level is low. For a given Brix level, a high ratio results in a large volume of hypertonic solution. Additionally, a high Brix level results in a greater amount of solute, leading to increased viscosity. This heightened viscosity gradually restricts the mobility of the hypertonic solution within the porous structure, resulting in surface saturation of the material with solute. Ultimately, this saturation forms a barrier that hinders the movement of water molecules from the material toward the hypertonic solution [24,62].

Furthermore, a low ratio necessitates less water to decrease the viscosity of the hypertonic solution, whereas a high ratio requires more water for viscosity reduction. Therefore, as the Brix level of the hypertonic solution decreases, the solute concentration diminishes more quickly. This rapid viscosity reduction not only facilitates infiltration and mobility of the osmotic solution within the material's porous structures but also promotes contact between the water-laden cells, aiding the removal of water from the core of the material toward the hypertonic solution.

So, when the Brix level is low, the viscosity of the hypertonic solution allows it to quickly penetrate through the porous structure to the core of the biological material. As the ratio increases, the decrease in the concentration of the hypertonic solution during osmotic dehydration (OD) becomes smaller, and the driving force that facilitates water removal from the material decreases gradually. In essence, the driving force provided by the hypertonic solution diminishes rapidly with a low ratio and decreases more slowly with a high ratio. If the driving force diminishes slowly, the biological material continues to lose water until equilibrium is achieved. This explains why we observe in Fig. 7 that WL increases with an increase in ratio when the Brix level is low.

When the Brix level is high, the viscosity of the hypertonic solution also increases. With a higher ratio under these conditions, the material's surface saturates more quickly with solute. The more saturated the surface becomes, the more challenging it becomes for the hypertonic solution to penetrate the core of the biological material and for water molecules to exit the material. This is why we observe a decrease in water loss (WL) with an increasing ratio when the Brix level is high. These observations differ from those reported by da Conceição Silva et al. (2012) [63] during osmotic dehydration (OD) of West Indian cherry, where they found that water loss, solid gain, and weight loss increased with increasing ratios. The discrepancy between our results and theirs could be closely related to the different structures of the two materials.

##### 3.1.4.2 Effects of the ratio-Brix interaction on solid gain (SG) during the D2I process

**Fig. 8:**
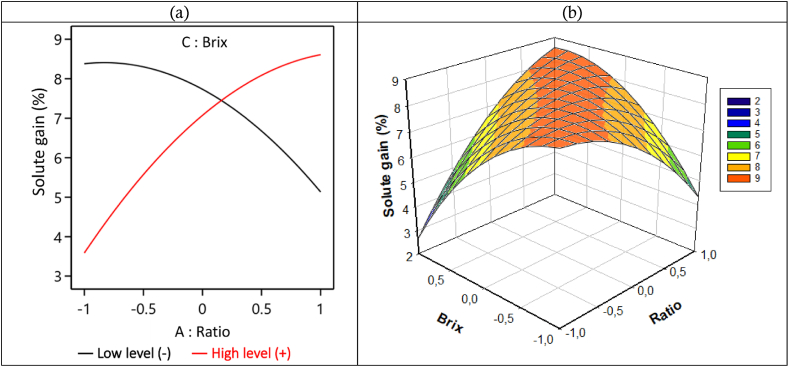
Interaction curve V/m ratio – Solution Brix degree on SG in (a) 2D and (b) 3D, during the D2I process.

Fig. 8 shows the effect of the interaction between the ratio V/m and the hypertonic solution concentration on the SG, during 240 min of immersion. From this figure, we observe that when the Brix is at its low level (−1 : 45°Brix), the SG decreases from 8.4 to 5 g solute/100 g (w-b) with the increase in the ratio from −1 to 1 (6/1 to 13/1 mL/g). But when the Brix is at its highest level (65 °Brix) we note an increase in SG as the ratio increases. Based on this analysis, it is clear that solid gain (SG) is minimal when the ratio is at a low level (6/1 mL/g) and Brix is at a high level (1 : 65 °Brix).

To understand why solid gain (SG) decreases with increasing ratio when the Brix is low and increases with increasing ratio when the Brix is high, we can relate it to what was discussed in the previous section.

When the Brix level is low, the viscosity of the hypertonic solution is also low, allowing it to easily infiltrate and move through the biological matrix. Additionally, a lower ratio results in less pressure exerted by the osmotic solution on the material, which further facilitates the infiltration and mobility of the hypertonic solution within the material. Conversely, with a higher Brix level, the viscosity of the hypertonic solution increases, making infiltration and movement through the material more challenging. A higher ratio increases the pressure exerted by the hypertonic solution, further reducing its infiltration into the material. As a result, when the solution can easily infiltrate and move through the material, more solute is adsorbed or adheres to the material. This could explain why SG is higher with a low ratio and decreases as the ratio increases.

When the Brix level is high and the ratio is low during osmotic dehydration (OD), the water leaving the material rapidly lowers the viscosity of the hypertonic solution, considerably slowing down the impregnation process. In contrast, with a high ratio, the Brix level of the solution does not fall quickly, and the water leaving the biological material does not rapidly lower the viscosity of the hypertonic solution. This results in rapid impregnation of the solute on the surface of the biological material. Under these conditions, the impregnation process dominates over the dehydration process. This could explain why solid gain (SG) is low at a low ratio when the Brix level is high and why SG increases with an increase in the ratio when the Brix level is high. These observations are similar to the results of da Conceição Silva et al. (2012) [63], who showed that water loss, solid gain, and weight loss increased during dehydration and were higher at increasing ratios. However, they found that an osmotic solution at a fruit/solution ratio of 10/1 mL/g was the best configuration studied, whereas our study identifies a ratio of 6/1 mL/g as the best configuration.

In line with the previous analyses on WL, we conclude that to have maximum WL and minimum SG, the D2I process should be carried out with a low ratio and high Brix. The multicriteria optimization will conduct us on the expected values.

### 3.2 Doehlert design results for mango slice treatment using the D3I process

#### 3.2.1 Experimental results, model expressions, and validation for the D2I process

The experimental matrix and the experimental and predicted WL and SG values are presented in Table 9 and it could be observed that the WL varies from 17.239 ± 0.815 % to 45.212 ± 1.038 %, whereas the SG varies from 1.193 ± 0.411 % to 5.668 ± 0.160 % according to the operating conditions predicted by the experimental matrix. From these results, we note that, with de-immersion during osmotic dehydration, we can achieve 45 % (nearly 50 %) WL and a maximum SG of less than 6 % (5.6 %), which already meets our expectations, given that we have opted for a WL of 50 % and an SG of 5 %.

**Table 9:** Experimental water loss (WL) and solute gain (SG) values, for the D3I process based on the Doehlert design.

	Factors			WL (g/100 g (w-b))		SG (g/100 g (w-b))	
Essay	X_1_ (min)	X_2_ (min)	X_3_ (min)	Exp	SD	Exp	SD
1	40	180	16	33.320	0.353	4.432	0.154
2	60	180	16	35.188	0.386	5.346	0.004
3	50	284	16	40.002	0.573	4.602	0.679
4	30	284	16	45.212	1.038	1.193	0.411
5	20	180	16	30.146	0.851	2.780	0.366
6	30	76	16	17.239	0.815	2.579	0.221
7	50	76	16	18.175	0.675	2.674	0.264
8	40	180	16	32.046	0.711	4.286	0.484
9	30	215	23	34.662	0.383	1.010	0.330
10	50	145	9	29.226	0.573	3.815	0.497
11	30	145	9	31.013	0.388	5.668	0.160
12	50	215	23	37.925	0.566	3.642	1.449
13	40	111	23	25.154	0.422	3.517	0.761
14	40	249	9	43.638	0.711	4.771	0.372
15	40	180	16	33.505	0.186	5.093	1.203
16	40	180	16	32.517	0.697	5.373	1.226

With Exp: experimental; SD: standard deviation, and X_1_, X_2_: and X_3_: real values of the factors, namely, immersion time, process time, and de-immersion time respectively.

The expressions of the models, in coded values, are given by equations (25), (26). All coefficient values, along with the characteristic quantities from the analysis of variance, are presented in Table 10.

(25) $$Y_{\text{WL}}=32.847+0.911x_{1}+14.341x_{2}-1.259x_{3}-0.180x_{1}^{2}-3.527x_{2}^{2}+2.064x_{3}^{2}-3.548x_{1}x_{2}+4.351x_{1}x_{3}-2.864x_{2}x_{3}$$

(26) $$Y_{\text{SG}}=4.796+1.177x_{1}-0.050x_{2}-1.243x_{3}-0.733x_{1}^{2}-2.468x_{2}^{2}-0.788x_{3}^{2}+1.913x_{1}x_{2}+2.071x_{1}x_{3}-1.476x_{2}x_{3}$$

where Y_WL_, Y_SG_, x_1_, x_2,_ and x_3_ are water loss, solute gain, immersion time, process time, and de-immersion time respectively.

**Table 10:** ANOVA of polynomial models predicting water loss (WL) and solute gain (SG) of mango slices in the D3I process.

Source	df	WL				SG			
		SCar	Contri (%)	Coef	P value	SCar	Contri (%)	Coef	P value
**Model**	9	879.666		32.847	0.000	28.431		4.796	0.009
**Linear**	3	832.232	49.964	/	0.000	11.719	20.721	/	0.009
Tps_Im	1	3.318	2.756	0.911	0.306	5.539	9.873	1.177	**0.009**
Tps_Proces	1	822.577	43.397	14.341	**0.000**	0.010	0.422	−0.050	0.876
Tps_De-Im	1	6.336	3.811	−1.259	0.173	6.169	10.426	−1.243	**0.007**
**Quadratique**	3	26.699	17.464	/	0.080	7.630	33.469	/	0.020
Tps_Im^2^	1	0.728	0.545	−0.180	0.903	0.399	6.152	−0.733	0.220
Tps_Proces^2^	1	19.601	10.672	−3.527	**0.047**	6.298	20.702	−2.468	**0.004**
Tps_De-Im^2^	1	6.370	6.248	2.064	0.172	0.932	6.615	−0.788	0.170
**Interaction**	3	20.736	32.572	/	0.147	9.081	45.810	/	0.017
Tps_Im*Tps_Proces	1	4.475	10.737	−3.548	0.108	5.206	16.053	1.913	**0.037**
Tps_Im*Tps_De-Im	1	11.343	13.167	4.351	**0.084**	2.569	17.375	2.071	**0.041**
Tps_Proces*Tps_De-Im	1	4.918	8.668	−2.864	0.222	1.306	12.382	−1.476	0.114
**Error**	6	15.927	/	/	/	2.298	/	/	/
Lack of fit	3	14.519	/	/	0.043	1.485	/	/	0.317
Pure error	3	1.408	/	/	/	0.813	/	/	/
**Total**	**15**	**895.593**	**100.00**	**/**	**/**	**30.730**	**100.00**	**/**	**/**

With df: degree of freedom; Coef: Coefficient; Contri: contribution and Lack of fit: Inadequacy of the fit; SCar: sum of squares of deviations.

The models obtained were evaluated using the validation criteria presented in equations (9), (10), (11), (12), (13). The values of these validation criteria are presented in Table 11 and show that for the WL and the SG models, all the statistic parameters (R^2^, adjusted R^2^, AADM, Bf, and Af), comply with the validation ranges presented in Table 5.

**Table 11:** Statistical values for the validation of WL and SG models in the D3I process.

Responses	R^2^	R^2^ adj	AADM	B_f_	A_f_
Y_SG_	92.52	81.30	0.096	0.997	1.079
Y_WL_	98.22	95.55	0.015	1.001	1.015

In the ANOVA table (Table 10), the p-value (0.317) for the lack-of-fit of the GS model indicates that there is no lack of fit. Therefore, the chosen model fits the data correctly, and the differences between the observed and predicted values are purely random and do not show any systematic trend; all variations observed in the SG data can be explained by the model. These results allow us to validate the quadratic model of equation (26) for the SG.

Regarding the WL, the ANOVA table (Table 10) presents a p-value of 0.043, indicating that the lack of fit is statistically significant at the 0.05 significance level. While the p-value for the lack of fit is an important factor, the other indicators (R^2^, adjusted R^2^, AADM, Bf, and Af) suggest that the SG model is overall strong. Indeed, the obtained high value of R^2^ of 0.98 means the model explains 98 % of the variance in the data; the value for the adjusted R^2^ of 0.95 indicates that even accounting for the number of variables in the model, it remains highly explanatory. The bias and accuracy are very close to 1 (1.001 and 1.015, respectively), showing that the model is accurate and unbiased, which is a good indicator of model quality. Also, the small absolute mean deviation value (0.015) indicates that the model's predictions are, on average, close to the observed values, which is another good thing for the model quality. Taking all these aspects into account, it appears that the model is overall good and robust. This is why the WL model is validated.

These validated WL and SG models were used to determine the predicted WL and SG values.

#### 3.2.2 Analysis of the significance and contribution of factors in the D3I process

The significance of the factors for each response was determined by performing an analysis of variances. The significance of the factors on the responses and the contribution of coefficients of each factor are presented in Table 10. In the case of WL, the contribution of factors with a simple effect is 49.96 % (almost 50 % contribution), that of factors with a quadratic effect is 17.46 %, and that of factors with an interaction effect is 32.57 %. Concerning SG, the contribution of simple-effect factors is 20.72 %, that of quadratic-effect factors is 33.47 %, and that of interaction-effect factors is 45.81 % (almost 50 % contribution).

Regarding the significance of the factors on the responses, Table 10 shows that Tps_Proces and its quadratic effect are the only parameters in the model that are significant at the 5 % threshold. However, it can be observed that the Tps_Im and Tps_De-Im interaction is significant at the 10 % threshold. In addition, the increase in Tps_Proces and the combination of Tps_Im and Tps_De-Im during the D3I have a positive effect on the WL, i.e. they increase the WL more (as being significant) than the other parameters during the D3I, whereas the quadratic effect of time has a negative effect on the WL, which leads us to understand that if the process time is very high, we will see a progressive decrease in the WL during the D3I.

Table 10 also shows that the quadratic effect (double effect) of Tps_Proces, Tps_De-Im, Tps_Im, the interaction between Tps_Im and Tps_Proces and the interaction between Tps_Im and Tps_De-Im are significant at the 5 % level. Furthermore, the quadratic effect of Tps_Proces and the increase in Tps_De-Im during D3I have a negative effect on SG, i.e. the higher the Tps_De-Im and the higher the Tps_Proces, the lower the SG, whereas the higher the Tps_Im, the combination of Tps_Im and Tps_Proces, and the combination of Tps_Im and Tps_De-Im have a positive effect on the SG. This generally means that the longer the mango slices are immersed in the hypertonic sucrose solution, the more they will absorb the solute, thus increasing the SG as the process time increases.

#### 3.2.3 Singular effects of factors on responses during the D3I process

##### 3.2.3.1 Singular effects of factors on water loss (WL) during the D3I process

**Fig. 9:**
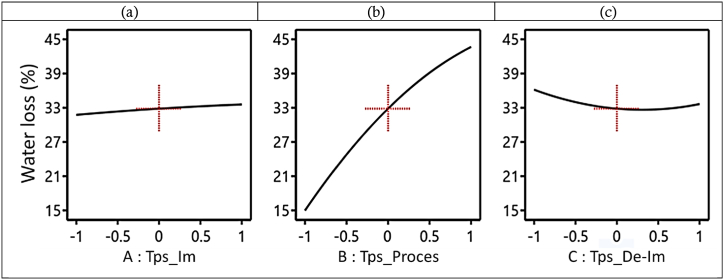
Evolution of WL as a function of (a) immersion time, (b) process time and (c) de-immersion time, during the D3I process.

Fig. 9 shows the evolution of WL as a function of immersion time (Tps_Im), D3I process time (Tps_Proces), and de-immersion time (Tps_De-Im). Fig. 9 (a) shows the evolution of the WL as a function of the immersion time. We can observe that the WL increases slightly in a straight line with immersion time, from 30 to 35 g of water/100 g (w-b). More precisely, for a process time of 180 min (3 h) and a de-immersion time of 16 min, the WL hardly varies for an immersion time of 20–60 min. However, in the case of intermittent dehydration in hypertonic solutions, long immersion times do not increase the WL.

In the case of the evolution of the WL as a function of the de-immersion time (Fig. 9 (c)) we also observe a slight decrease in the WL from 37 to 33 g of water/100 g (w-b) and a very slight increase in the WL from 33 to 35 g of water/100 g (w-b). In other words, for a process time of 180 min (3 h) and an immersion time of 40 min, the WL hardly varies for a de-immersion time of 7–25 min. We can say at the outset that the increase in de-immersion does not favor an increase in WL during D3I. The simple effects of immersion time and de-immersion time on WL, during 180 min, are barely perceptible but, on the other hand, in Fig. 9 (b), we realize that the longer the process time, to be more precise, the more the number of intermittences (the repetition of the immersion/de-immersion operation) increases during the D3I process, we see an increase in the WL from 15 to 45 g of water/100 g (w-b) for a process time ranging from −1 to 1 (60–300 min) for an immersion time of 40 min and a de-immersion time of 16 min. We can see that not only can we achieve 45 % WL with D3I, as with D2I, but it is also possible to have operating conditions with a maximum WL of 50 %.

As in the D2I process, the WL in the D3I process increases with the duration of the process, but the WL is slightly higher in D2I than in D3I. This is because when the immersion of the biological material is interrupted for a certain period, there is a slowing down of the material transfer processes from the surface of the material to the interior (solute) and from the interior of the material to the surface of the product, then to the hypertonic solution (water), which would explain why the WL is slightly lower in the D3I process than in the D2I process.

##### 3.2.3.2 Singular effects of factors on solute gain (SG) during the D3I process

**Fig. 10:**
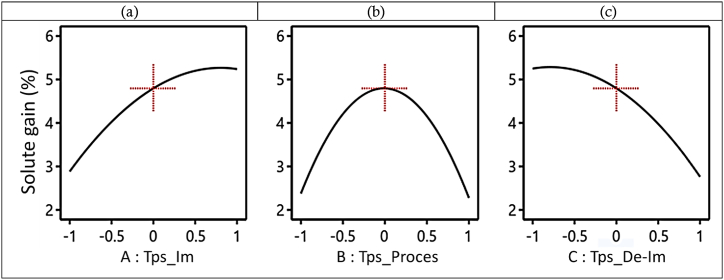
Evolution of SG as a function of (a) immersion time, (b) process time and (c) de-immersion time, during the D3I process.

Fig. 10 shows the evolution of SG as a function of immersion time (Tps_Im), D3I process time (Tps_Proces), and de-immersion time (Tps_De-Im). Fig. 10(a) illustrates the progression of SG as a function of immersion time, demonstrating an increase from 2.9 to 5.4 g solute/100 g (w-b) with increasing immersion time, peaking at 60 min. In the case of the evolution of SG as a function of de-immersion time (Fig. 10(c)), SG decreases (from 5.4 to 2.9 g solute/100 g (w-b)) with increasing de-immersion time. Therefore, it can be concluded that the longer the mango slices remain immersed in the hypertonic sucrose solution, the more they adsorb and absorb the sucrose solution, and the less sucrose adsorption and absorption of the hypertonic solution are observed. We observe that shorter immersion times and longer de-immersion times lead to lower solute gain (SG) during D3I in the mango slice matrix.

Fig. 10 (b) shows the evolution of the SG as a function of the process time. This figure indicates that the SG increases from −1 to 0 (60–80 min), reaches a maximum at the coded value of 0 (180 min), and then decreases from 0 to 1 (180–300 min). During the immersion and de-immersion of mango slices in the hypertonic solution, we can conclude that shorter process times result in high SG values, whereas longer process times result in low SG values. On the other hand, during the D2I process, where we have continuous immersion, we observe a progressive increase in SG until equilibrium is reached. SG values can go up to 10 or 15 g solute/100 g (w-b) [24,61].

During the D3I process, the exudation phenomena during de-immersion slow down the mechanisms of sucrose adsorption and hypertonic solution absorption. In the absence of a hypertonic solution, the rate of matter transfer is abruptly reduced, and repeated interruptions of this transfer eventually hinder the mechanisms that allow SG to increase. These exudation phenomena occurring in the mango slices are driven by gravity, which causes the hypertonic micro-solutions within the pores to exit, carrying water molecules from the mango slices with them. This reduces not only the water content but also the solute content in the material. Consequently, as the de-immersion time increases, the SG decreases, and the more frequently the de-immersion operation is repeated, the further the SG decreases.

To better understand the interplay between immersion and de-immersion during the dehydration process in hypertonic solutions, interaction curves have been drawn and are presented in the following section 3.2.4.

#### 3.2.4 Effects of immersion time – de-immersion time interaction on responses during D3I

**Fig. 11:**
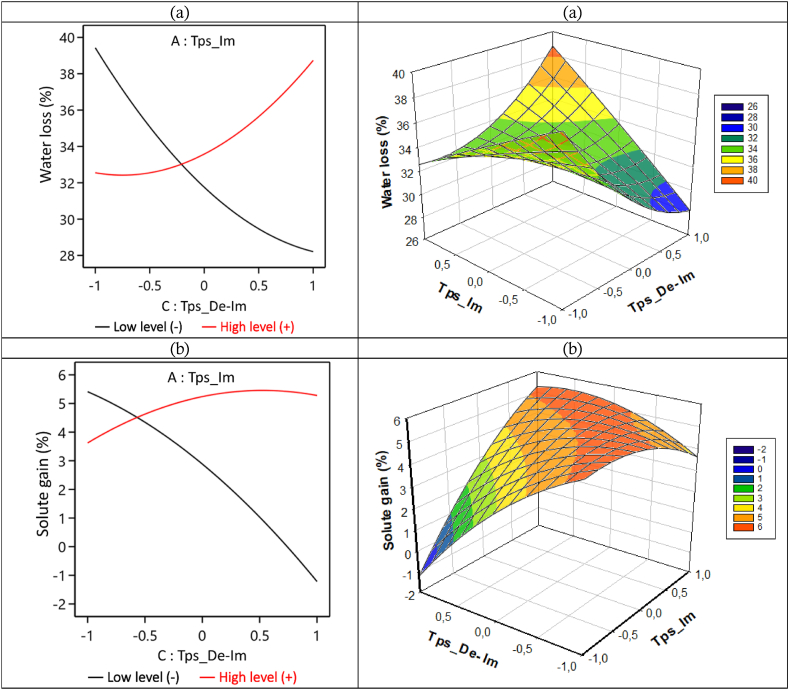
Immersion time (Tps_Im) – de-immersion time (Tps_De-Im) interaction curve for WL (a) and SG (b), during the D3I process.

Fig. 11 (a) and Fig. 11 (b) respectively show the evolution of WL and SG as a function of the interaction between immersion time and de-immersion time for an osmotic treatment of 180 min. These curves show that when the immersion time is at its low level (−1 : 20 min) the WL and SG decrease, respectively from 39.5 to 28 g of water/100 g (w-b) and from 5.5 to −1 g of solute/100 g (w-b), with the increase in de-immersion time going from −1 to 1 (7 min–25 min). In other words, when the immersion time is short and the biological material remains out of the hypertonic solution more and more, the transfer of matter also slows down more and more.

From the same curves, it can be observed that when immersion time is at its high level (1 : 60 min) the WL and SG increase respectively from 32.5 to 39 g of water/100 g (w-b) and from 3.5 to 5.5 g of solute/100 g (w-b) with the increase in de-immersion time going from −1 to 1 (7 min–25 min). In other words, when the immersion time is high and the biological material remains outside the hypertonic solution more and more, the transfer of matter does not slow down because there is still an increase in WL and SG. Even so, we can assume that although there is no net slowdown as above, the de-immersion has contributed in some way to slowing down the matter transfers.

Based on these analyses, we can assert that when the immersion time is short, de-immersion significantly slows down matter transfer to the point of causing a decrease in WL and SG, but when the immersion time is long, de-immersion slows down but not to the point of significantly slowing down matter transfer, as we still observe an increase in WL and SG. We can observe (which is to be expected) that de-immersion slows down matter transfer during osmotic dehydration but does not stop it. Indeed, de-immersion will not enable us to reach a maximum WL quickly, but it will nevertheless enable us to slow down the impregnation process as much as possible, resulting in a minimum SG at the end of the impregnation process.

In summary, to obtain maximum WL and minimum SG, osmotic dehydration should ideally be carried out under conditions of short immersion time, short de-immersion time, and long processing time.

### 3.3 Optimal conditions determination for D2I and D3I processes

Following data analysis of the D2I and D3I processes, we conducted multi-criteria optimization on each process using the desirability function in Minitab 21 software.

Concerning the D2I process, the goal was to minimize SG and maximize WL. Table 12 presents the top seven optimal predicted solutions for the D2I process. This table shows that solution 1, with a desirability of 1, meets the validation criteria specified by the desirability function. Therefore, solution 1 was chosen as optimal. The coded and uncoded values for this optimal point for the D2I process are shown in Table 13.

**Table 12:** Top seven optimal predicted solutions for the D2I process.

Solution	Ratio V/m (-)	Time (-)	°Brix (-)	WL (g/100 g (w-b)	SG (g/100 g (w-b))	Desirability (-)
**1**	**−1.000**	**0.0372**	**0.6565**	**50.000**	**5.000**	**1.000**
2	−1.000	−0.0065	0.5013	48.884	5.000	0.981
3	−0.456	−0.2950	0.4187	42.181	5.978	0.671
4	1.000	0.7499	−0.6219	43.346	5.001	0.611
5	1.000	0.7499	−0.62189	43.347	5.001	0.611
6	−0.976	0.7499	−0.5075	39.807	7.438	0.599
7	−1.000	0.7499	0.1957	51.706	7.946	0.587

**Table 13:** Optimal coded and uncoded predicted factor values and response values for the D2I and D3I processes.

	D2I Process			D3I Process		
Nature of the physical quantity	Ratio V/m (g/ml)	Time (min)	Brix (g/100 g)	Tps_Im (min)	Tps_Proces (min)	Tps_De-Im (min)
**Coded values (no unit)**	−1	0.0372	0.6565	−0.966	0.7805	−0.816
**Uncoded values (with unit)**	6/1	250	61.6	21	270	9
Optimal predicted responses						
**WL**	50				52,43	
**SG**		5			4	

**Fig. 12:**
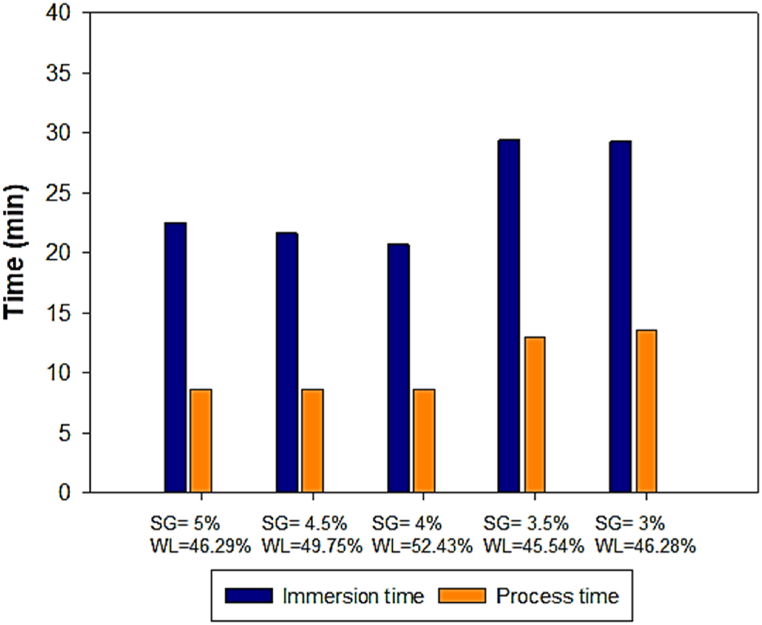
Best times giving minimum SG and maximum WL, for the D3I process.

About the D3I process, the multi-criteria optimization was conducted through five optimizations, by targeting solute gain at specific values below 5 % (5, 4.5, 4, 3.5, and 3), as these represent the range of the lowest values observed during this study, and then maximizing water loss for each case. Subsequently, the optimization process yielded the respective optimal times for immersion and de-immersion for each case. All initial solutions generated by the software were selected due to their high desirability scores (all ranging between 0.988655 and 1), compared to other subsequent solutions. These predicted optimal responses are plotted in Fig. 12, along with the optimal operating conditions for each targeted solute gain value.

To select the best candidate among these five optima, immersion time (Tps_Im) and de-immersion time (Tps_De-Im) were utilized. According to the curve analysis showing the interaction between immersion and de-immersion times, achieving high water loss requires working with lower immersion and de-immersion times for a longer process duration. From these different optimal points presented in Fig. 12, the optimal process time varies between 235 and 285 min.

Based on the analyses in the previous sections (3.1. and 3.2), to achieve a low SG and a high WL, both the immersion and de-immersion times need to be minimized while maintaining a high process time. We have chosen the following optimal point (SG = 4 % and WL = 52 %), which corresponds to 270 min of process time, with the shortest immersion and de-immersion times. Indeed, the process time (Tps_Proces) is the sum of two components, namely the immersion time (Tps_Im) and the de-immersion time (Tps_De-Im). Given that the optimal values for these two components have been determined, it follows that the optimal process time is 30*9 = 270 min, where 30 is the sum (21 + 9) min corresponding respectively to the optimal immersion time (Tps_Im) and de-immersion time, and 9 is the number of cycles at optimum. The coded and uncoded values for this optimum for the D3I process, are presented in Table 13. This Table indicates that the D3I process has a longer optimal predicted process time compared to that of the D2I process, whereas the optimal predicted solute gain obtained on the D2I process is greater than that on the D3I process.

### 3.4 Statistical tests on the optimal predicted and experimental response values

Laboratory tests were performed using the obtained optimal operating conditions, and the results of the experimental responses are presented in Table 14, along with the corresponding predicted optimal values.

**Table 14:** Student's t-test comparing predicted and experimental optimal water loss (WL) and solute gain (SG) for the D2I and D3I processes.

	WL (D2I)		SG (D2I)		WL (D3I)		SG (D3I)	
**Nature of the physical quantity**	Optimal predicted responses	Optimal experimental responses	Optimal predicted responses	Optimal experimental responses	Optimal predicted responses	Optimal experimental responses	Optimal predicted responses	Optimal experimental responses
**DF, F-test = (n**–**p)**	5		5		6		6	
**t**_**alpha/2, (n-p)**_	2.57		2.57		2.45		2.45	
**MSE**	0.57		0.42		2.65		0.38	
**SEP**	0.75		0.65		1.63		0.62	
**Mean**	50.00 ± 1.94	47.63 ± 1.79	5.00 ± 1.66	6.67 ± 1.04	52.43 ± 3.99	47.98 ± 2.12	4 ± 1.51	4.31 ± 0.54
**Variance**	3.76	3.20	2.76	1.08	15.92	4.49	2.28	0.25
**F-statistic**	1.17		2.55		3.54		7.82	
**F-critical**	19		19		19		19	
**p-value of the F-statistic**	**0.92**		**0.56**		**0.44**		**0.23**	
**Decision for homoscedasticity**	Accept the equality of variances		Accept the equality of variances		Accept the equality of variances		Accept the equality of variances	
**Pooled standard deviation**	3.50		2.09		11.70		1.63	
**t-statistic**	0.83		0.98		0.47		0.23	
**Degree of freedom**	4		4		4		4	
**t-critical (a = 0.05)**	2.78		2.78		2.78		2.78	
**p-value of the t-statistic**	**0.19**		**0.21**		**0.16**		**0.75**	
**Decision for the *t*-test**	Accept H0		Accept H0		Accept H0		Accept H0	
**D2I versus D3I predicted optimal responses**	O* for WL (D2I) versus WL (D3I) = (4/0.26/2.78//**0.40**/0.05) (F-test p-value = 0.38)							
	O* for SG (D2I) versus SG (D3I) = (4/0.48/2.78//**0.48**/0.05) (F-test p-value = 0.91)							
**D2I versus D3I experimental optimal responses**	O* for WL (D2I) versus WL (D3I) = (4/0.11/2.78//**0.84**/0.05)							
	O* for SG (D2I) versus SG (D3I) = (4/3.68/2.78//0.02/0.05)							
**Notation**	H0: There is no significant difference between the mean of the optimal experimental responses and the optimal predicted response.							
	H1: There is a significant difference between the mean of the optimal experimental responses and the optimal predicted response.							
	O* = (degree of freedom/t-statistic/t-critical//p-value of the t-statistic/significance level); SEP: standard error of prediction.							

The Fisher test, applied to the predicted optimal responses and the experimental ones, shows that they have the same variance, as all the p-values obtained are greater than the significance level of 5 % (Table 14). Therefore, the pooled *t*-test was performed to compare the means of these two groups. The calculations were performed using the equations presented in section 2.7.

The results of the t-tests at the 5 % significance level are presented in Table 14. Regarding the comparison between the predicted optimal values and the experimental values obtained for each process, Table 14 shows no significant difference at the 5 % significance level between the predicted optimum and the experimental optimum of WL for the D2I process. The same conclusion applies to the comparison of the predicted optimal SG and the experimental SG optimum for the D2I process. The D3I process presents similar results for the comparison of predicted and experimental optimal transfer quantities.

A *t*-test was also conducted to compare the two processes, through their mean transfer physical quantities. The hypothesis of homoscedasticity for the variances was validated by a Fisher test, as shown in Table 14. Among all the pairwise comparisons of responses, only the test comparing the experimental optimal solute gain responses of the two processes rejected the null hypothesis, indicating sufficient evidence of a difference between these two sets of experimental data. This significant difference in the experimental optimal means of the D2I and D3I processes is noteworthy, as it underscores the advantages of the D3I over the D2I process. Specifically, the D3I process results in a product with a SG that is 1.5 times lower compared to the D2I process, especially with longer process times (greater than 4 h).

Finally, these results demonstrate that the experimental validation of the models' optima has been successfully achieved and that the models determined for each response and each process (D2I or D3I), can be used to predict the behavior of mango slices subjected to these processes.

## 4 Conclusion

The objective of this study was to determine the optimal conditions for processing mango slices, from the “café” variety, using D2I and D3I processes, aimed at maximizing water loss and minimizing solute gain. The analysis of variance, optimization using the desirability function, and statistical tests underscored the importance of reducing the V/m ratio to maximize water loss (WL) and minimize solute gain (SG). The optimal D2I treatment conditions correspond to a ratio of 6/1 (mL/g), an immersion time of 245 min, and a solute concentration of 61.6 (g/100 g). These optimum conditions resulted in a water loss (WL) of 47.626 ± 1.793 % (g/100 g) and a solute gain (SG) of 6.67 ± 1.04 % (g/100 g), helping to reduce the initial water content by 25.36 %. Concerning the D3I process, the best optimum times for treating mango slices correspond to an immersion time of 21 min, a de-immersion time of 9 min, and a process time of 270 min. These optimum conditions resulted in a water loss (WL) of 48.0 ± 2.1 % (g/100 g) and a solute gain (SG) of 4.3 ± 0.1 % (g/100 g). The multiple analyses carried out show that repeated immersion and de-immersion sets, lead to an increase in WL and limit SG. Based on the results of solute gain and water loss obtained through experimental design modeling, intermittency during osmotic dehydration of mango slices could be effectively employed as a pre-treatment before hot-air drying, rather than using a continuous immersion process, especially for longer immersion durations. Experimental design models may not always fully integrate theoretical principles. Conducting kinetic studies using semi-empirical models, which blend theoretical principles with empirical data, would enhance understanding of complex systems like those in osmotic dehydration, where purely theoretical or empirical approaches may not suffice. Furthermore, to better understand the impact of intermittent immersion during D3I, it is crucial to evaluate changes in the physical, nutritional, organoleptic, and functional properties of mango slices, as well as to assess the efficacy and energy efficiency of this new process and compare it with other dehydration techniques.

## Funding

This research was not funded.

Regarding the publication procedure, the authors are supported by Heliyon, which covers 100 % of the Article Processing Charges (APC). The corresponding author and the co-author, both from a Group A country (Cameroon), are thankful for this support.

## Statement for acceptance of the publication of this work

⁃ All the authors approve the publication of this work.
⁃ The head of the LARESH Laboratory, where the work was carried out approves the publication of this work.

## Animal and human experiments

The present work doesn't include animal or human experiments.

## Data availability statement

⁃ Data associated with our study were not deposited into a publicly available repository.
⁃ The data are included in the article and are accessible to the public. Further information will be made available upon request.

## CRediT authorship contribution statement

**C. Tsopwo Zena:** Writing – original draft, Methodology, Investigation, Funding acquisition, Formal analysis, Data curation. **Y. Jiokap Nono:** Writing – review & editing, Writing – original draft, Visualization, Validation, Supervision, Resources, Project administration, Methodology, Investigation, Funding acquisition, Formal analysis, Data curation, Conceptualization.

## Declaration of competing interest

The authors declare that they have no known competing financial interests or personal relationships that could have appeared to influence the work reported in this paper.
